# Effect of biological soil crusts on seed germination and growth of an exotic and two native plant species in an arid ecosystem

**DOI:** 10.1371/journal.pone.0185839

**Published:** 2017-10-04

**Authors:** Guang Song, Xinrong Li, Rong Hui

**Affiliations:** 1 Shapotou Desert Research and Experimental Station, Northwest Institute of Eco-Environment and Resources, Chinese Academy of Science, Lanzhou, China; 2 University of Chinese Academy of Sciences, Beijing, China; US Geological Survey, UNITED STATES

## Abstract

Biological soil crusts (BSCs) can improve the stability and health of native plant communities in arid ecosystems. However, it is unknown whether BSCs can also inhibit invasions of exotic vascular plants on stabilized reclaimed sand dunes. To answer this question, we conducted a greenhouse experiment to test the effects of cyanobacteria-dominated BSCs on 1) seed germination and biomass of an exotic grass (*Stipa glareosa* P. Smirn.), and 2) individual biomass of the exotic *S*. *glareosa* growing with two native plants, *Eragrostis poaeoides* Beauv. and *Artemisia capillaris* Thunb. Our experiment included three BSC treatments (intact crust, disturbed crust, and bare soil) and five species trials (native *E*. *poaeoides* alone, *E*. *poaeoides* mixed with exotic *S*. *glareosa*, native *A*. *capillaris* alone, *A*. *capillaris* mixed with exotic *S*. *glareosa*, and *S*. *glareosa* alone). The results showed that cyanobacteria-dominated crusts can significantly reduce the cumulative percent germination of the exotic grass (P<0.001) and native plants (P<0.001). Maximum cumulative percent germinations of the exotic grass and two native plants were found in bare soil, and minimum in intact crusts. The interaction of crust treatment × species trials on shoot biomass of the two native plants was significant (*P*<0.05). These results indicate that the presence of BSCs on stabilized sand dunes may reduce the germination of the exotic and two native plants. The effect of reducing exotic and native plant seeds germination would maintain more diverse plant communities and contribute to the formation of clumped vegetation patterns. We conclude that BSCs act as a natural regulator for vegetation patterns and thus promote ecosystem stability and sustainability.

## Introduction

Drylands, areas with a severe and fragile ecological environment, cover more than one third of the terrestrial land surface [1,2]. Drylands are characterized by a sparse presence of vascular plants due to low water availability, and low rates of nutrient turnover or a limited ability to acquire resources due to extreme temperatures [3,4]. The soil surface in drylands is often occupied by biological soil crusts (BSCs) [5,6]. BSCs are considered “ecosystem engineers” of arid and semiarid lands [7,8] and indicators of ecosystem health, and are linked with the level of dryland ecosystems restoration or degradation of dryland ecosystems [9–11].They are typically composed of diminutive organisms such as cyanobacteria, algae, lichens, mosses, and other organisms, occurring in varying proportions [5,12]. BSCs create a boundary for most dissolved inputs and losses from soils [5]. They influence soil water distribution by affecting rainfall infiltration [13,14], decreasing soil evaporation [15–17], and absorbing condensation moisture [18,19]. In addition, BSCs may improve the stability of soil [20,21] via their ability to withstand erosion [22], and fix carbon and nitrogen [23–25].

The benefits of a more-favorable environment and increased availability of resources provided by BSCs may include higher rates of seed germination, and establishment, and improved performance of co-evolved native vascular plants [3,26,27]. Previous studies on the relationship between vascular plants and BSCs indicated that the effects of BSCs on these vascular plants may be negative, positive or uncorrelated [20,26,28–31]. Li et al. [32] found that survival and growth of the two native annual plants were enhanced in both algal and moss crusts during the season of rainfall or in moist environment in the Tengger Desert. Thiet et al. [33] evaluated the relationship between BSCs and two native dune plants in Cape Cod of Atlantic-facing coastal. They found that BSCs (particularly algal crusts and moss mats) may help plants establish and survive by providing safe microsites that buffer seedlings against drought and wind, but seedlings may then be forced to compete with crusts and mats for moisture and nutrients. Some other studies suggested that the effects of BSCs on vascular plants are species specific. Researches in desert of northern China found that BSCs did not affect percent germination in plants with smooth seeds (e.g. *Ephedra*. *distachya*), but inhibited germination of seeds with appendages (e.g. *Haloxylon persicum*) [34,35]. Godinez-Alvarez et al. [36] reported that cyanobacterial and a mixed moss–cyanobacterial crust have positive effects on the germination of *Agave marmorata*, but they have no effects on on either *Prosopis laevigata* or *Neobuxbaumia tetetzo* in a tropical desert of Mexico. For emerged seedling, BSCs may have positive effects on their survival and growth [5,34,36,37].

A study focusing on the relationships between BSCs and invasions of exotic vascular plants revealed that the interaction between disturbance (such as grazing) and detrimental non-native can decrease BSCs cover in Western Colorado [38]. DeCorte [39] found that soils covered by BSCs and by desert pavement (consisting mainly of rocky outcrops) have fewer exotic plants than found on open surface in the Mojave Desert. Belnap et al. [40] explored the relationships between the dynamics of BSCs and plant invasion by *B*. *tectorum*, precipitation, and temperature in semiarid grasslands in southeastern Utah, USA. They found that the cover of BSCs (lichens and mosses) in desert ecosystems decreased due to plant invasions, increasing air temperatures, and the timing of precipitation. Additionally, exotic species exhibited a greater emergence in BSCs disturbed by human footprints or grazing than in undisturbed BSCs in a California sage scrub [41]. These studies revealed a negative relationship between BSCs and exotic vascular plants that have not co-evolved [42,43].

It has long been known that exotic vascular plant invasions are a serious threat to natural and semi-natural ecosystems worldwide [44–46]. Particularly, arid ecosystems are vulnerable to plant invasions due to a low capacity for resisting disturbances, and low resilience [47,48]. As an example, natural xeric habitats of the Peri-Caribbean Arid Belt on the Caribbean coast of Colombia are classified as endangered partly due to the establishment of alien species [45]. Many evidences suggest that invasive plants can have strong effects on native plant species via both direct and indirect effects [49–51]. Flory et al. [52] quantified the direct effects of non-native invasive species on native species, they found that biomass of native plants were significantly reduced by non-native invasive species. Another study in Indiana, USA showed that invader *Microstegium* may be directly reducing native tree regeneration through competition, thus may further lead to slowing the rate of forest succession [53].

Research on plant invasions in arid areas concentrated to date on the relationships of exotic plants and nitrogen dynamics [47,54,55], changes in fire regimes [56–59] and native plant community composition and dynamics [60–62]. Because BSCs are the main biological cover in arid regions, they are considered to be a critical biotic factor in inhibiting germination and establishment of exotic vascular plants [26,41]. However, experimental evidence is not enough to support the possible mechanisms of BSCs prevention of exotic grass invasions.

In this study, we investigated the impacts of BSCs on an exotic and two native vascular plant species in the Shapotou re-vegetation area, located on the southeastern edge of the Tengger Desert, Ningxia Hui Autonomous Region. The main aim of re-vegetation was to protect the railway from blowing sands, and to ensure a normal operation of the railway. Consequently, a re-vegetation zone was established on both sides of the railway, with a length of 16 km and width of 200 to 1,000 m [63]. After more than 50 years, this re-vegetation zone not only ensured the safety of the railway operation, but also significantly improved the habitat for vascular plants. Once the sand dunes were stabilized by straw checkerboards combined with vegetation method, cyanobacterial crusts began colonizing the sand surface; these early-successional BSCs mainly included cyanobacteria, algae. Later, early-successional BSCs developed into lichen-, and moss-dominated crusts. After decades of vegetation development and colonization, the cover of BSCs in the re-vegetated zone surpassed that in the adjacent natural vegetation zone. For example, the cover of BSCs reached more than 90% in the lowlands between the dunes after 50 years of sand stabilization [64]. Accordingly, we wanted to determine whether cyanobacterial crusts limited exotic plant invasions. We conducted a greenhouse germination experiment to test the hypotheses that 1) the presence of cyanobacterial crusts decreases exotic vascular grass *S*. *glareosa* seed germination and biomass; and that 2) the exotic grass decreases the biomass of two native plants, but the effect may be weakened due to the presence of cyanobacterial crusts. Our aim was to investigate whether BSCs play an important role in preventing exotic plant invasions in the drylands, and to provide a scientific basis for BSCs inclusion in arid ecosystem management.

## Materials and methods

### Ethics statement

The study area belongs to the Shapotou Desert Research and Experimental Station, Chinese Academy of Sciences (37°33′N, 105°02′E), a department of the Northwest Institute of Eco-Environment and Resources, Chinese Academy of Science. The study was approved by the Northwest Institute of Eco-Environment and Resources, Chinese Academy of Sciences. The field work did not involve any endangered or protected species, and did not involve destructive sampling.

### Study site

The study area (37°33′N, 105°02′E) is situated in the Ningxia Hui Autonomous Region at the southeastern edge of the Tengger Desert in northern China; at an elevation of 1,339 m. Mean annual air temperature is 10.0°C, with a minimum of -25.1°C in January, and a maximum of 38.1°C in July. Mean annual precipitation is approximately 180.2 mm (1956–2012); about 80% of precipitation falls as rainfall from May to September, the annual potential evaporation is about 3000 mm. The annual duration of sunshine is 3,264 h, mean annual wind velocity is 2.9 m s^−1^, and the number of dust-storm days per year is 59 [63].

The area is a typical temperate desert. Soils are loose and nutrient-depleted, characterized by shifting sands and a moisture content consistently ranging from 3 to 4% [63]. The re-vegetation plant community is dominated by *Eragrostis poaeides*, *Artemisia capillaris*, *Setaria viridis*, *Caragana korshinskii*, *Atraphaxis korshinskii* and *Bassia dasyphylla* [15,65].

### Species selection and sampling

We selected *Stipa glareosa P*. *Smirn*. as the invasive exotic grass species because it possessed many important characteristics to survive in the Tengger desert, such as a well-developed root system, cold resistance, and extreme drought- and high- temperature tolerance. Most importantly, *S*. *glareosa* can produce a multitude of propagules which are easily dispersed by wind and by adhesion to other objects [66,67]. Additionally, anthropogenic disturbance, in particular, the collection of *Allium mongolicum* (collected for its unique edible and medicinal values) and the transport by railway and highway, may increase the probability of spread of *S*. *glareosa* seeds into the re-vegetation zone [68,69]. Recently, *S*. *glareosa* was found in the re-vegetated area [70], therefore we considered *S*. *glareosa* as a potential invasive exotic grass in the re-vegetation area. Seed of *S*. *glareosa* is a long strip or rod with length of 6–9 mm, a slender barb at the proximal embryo, awn 4.5–7 cm long, and pilose along its whole length.

*Eragrostis poaeoides* Beauv., and *Artemisia capillaris* Thunb., are the most common annual and perennial herbaceous species in the re-vegetation area; they were selected as the native plant species for this research. Seeds of the exotic and native grasses were collected during the time of maturation (May to June and September to October, respectively) in 2013, and kept at ambient temperature in the laboratory until beginning of the experiments. *E*. *poaeoides* seed are red-brown, oblong or globose, ca. 0.5 mm in diam and *A*. *capillaris*.seeds are brown, oblong-ovate, ca. 0.8 mm.

We collected one type of naturally-occurring BSCs in the summer of 2014 from the re-vegetation zone at the southeastern edge of the Tengger Desert in northern China. The collected BSCs were primarily composed of cyanobacteria, with lichens and mosses comprising a small fraction. We used a specially-made PVC sampler, 18 cm in diameter and 20 cm high, to collect crusts with the top 15–20 cm of soil to ensure sufficient soil depth for the growth of grass roots. To prevent soil and crust break-up, soil was kept slightly moist during collections. Samples of intact crusts constituted the intact treatment. For the disturbed-crust treatment, the collected cyanobacterial crusts were severed with a spade, and then partly mixed with the soil beneath (mimicking animal disturbance). The bare soil (reference) treatment did not contain cyanobacterial crusts, and bare soil was collected at the same site as the cyanobacterial crust samples, using the same method. The cyanobacterial crust treatments represented the main ecological scenarios: intact, partly-disturbed by soil animals or human activity, and bare soil without cyanobacterial crust.

Experimental pots (12 cm in diameter and 20 cm high) were filled with sand taken from a sand dune 0.3 m below the surface to avoid native seeds from the seed bank. Before the experiment started, all pots were placed in a greenhouse and sprayed with water to keep the soil surface moist. Pots were then observed for the emergence of remnant seeds. If any seedlings emerged, they were removed. Sand was considered seed-free if no new seedlings emerged for a period of 6 weeks. This pre-treatment aimed to eliminate the interference of the native seed bank in the subsequent experiment. After pre-treatment, samples of crusts were placed in the pots.

### Experimental design

We conducted the experiment in the greenhouse of the Shapotou Desert Research and Experimental Station, Chinese Academy of Sciences from July to October of 2014. Our experiments consisted of three cyanobacterial crust treatments (intact crust, disturbed crust, and bare soil), and five plant species-trials (three monocultures and two mixed-cultivation tests) resulting in fifteen treatment combinations (cyanobacterial crusts × plant species treatments). The three monocultures included the native species *E*. *poaeoides* and *A*. *capillaris*, and an exotic *S*. *glareosa*. The two mixed-cultivation tests included *E*. *poaeoides* with *S*. *glareosa*, and *A*. *capillaris* with *S*. *glareosa*. Each treatment combination was replicated five times for a total of 75 pots in the same greenhouse chamber.

In the three monoculture treatments, twenty seeds of *E*. *poaeoides*, *A*. *capillaris* or *S*. *glareosa* were sown per pot; in the two mixed-cultivation treatments, ten seeds of each *E*. *poaeoides* and *S*. *glareosa*, or *A*. *capillaris* and *S*. *glareosa* were sown per pot. To break dormancy of *S*. *glareosa* seeds, seeds were placed in a cryostat at 5°C for 4 weeks prior to sowing. Prior to the greenhouse experiments, we tested the seeds germination rates of exotic and native plant species on moist filter paper, and the germination rates were 82% for *S*. *glareosa*, 80% for *E*. *poaeoides* and 86% for *A*. *capillaris* (based on 20 seeds). After sowing, pots were checked every day for ca. 2.5 months, and the number of germinated seeds of each species was counted. We also calculated the time to 50% cumulative germination (T50) for each treatment of every species. The mean T50 between treatments determines a difference in seedling vigor as a measurement of growth speed [71]. Seeds were considered germinated when the radicle was visible. During the experiment, each pot was sprayed with 100 ml of distilled water at the same time each day. Shoots were harvested on October 26, 2014; each plant was clipped near the soil surface, and plants were separated by species. Aboveground biomass of individual plants was weighed after drying for 48 h at 65°C.

### Statistical analysis

Statistical analysis was performed using the SPSS statistical package (IBM SPSS software, 20^th^ version, Chicago, USA). We used two-way ANOVAs to test for species and crust treatment effects on the cumulative percent germination andshoot biomass of the exotic and native plants. The influence factors were cyanobacterial crusts treatments and species trials. Significant differences among treatments were determined using the Tukey test at *P* < 0.05. We also tested the difference of T50 values among treatments using the one-way ANOVAs. Because data on seed cumulative percent germination and shoot biomass did not follow a normal distribution, data were arcsine- and log-transformed, respectively.

## Results

### Influence of cyanobacterial crusts on the germination

**Fig 1:**
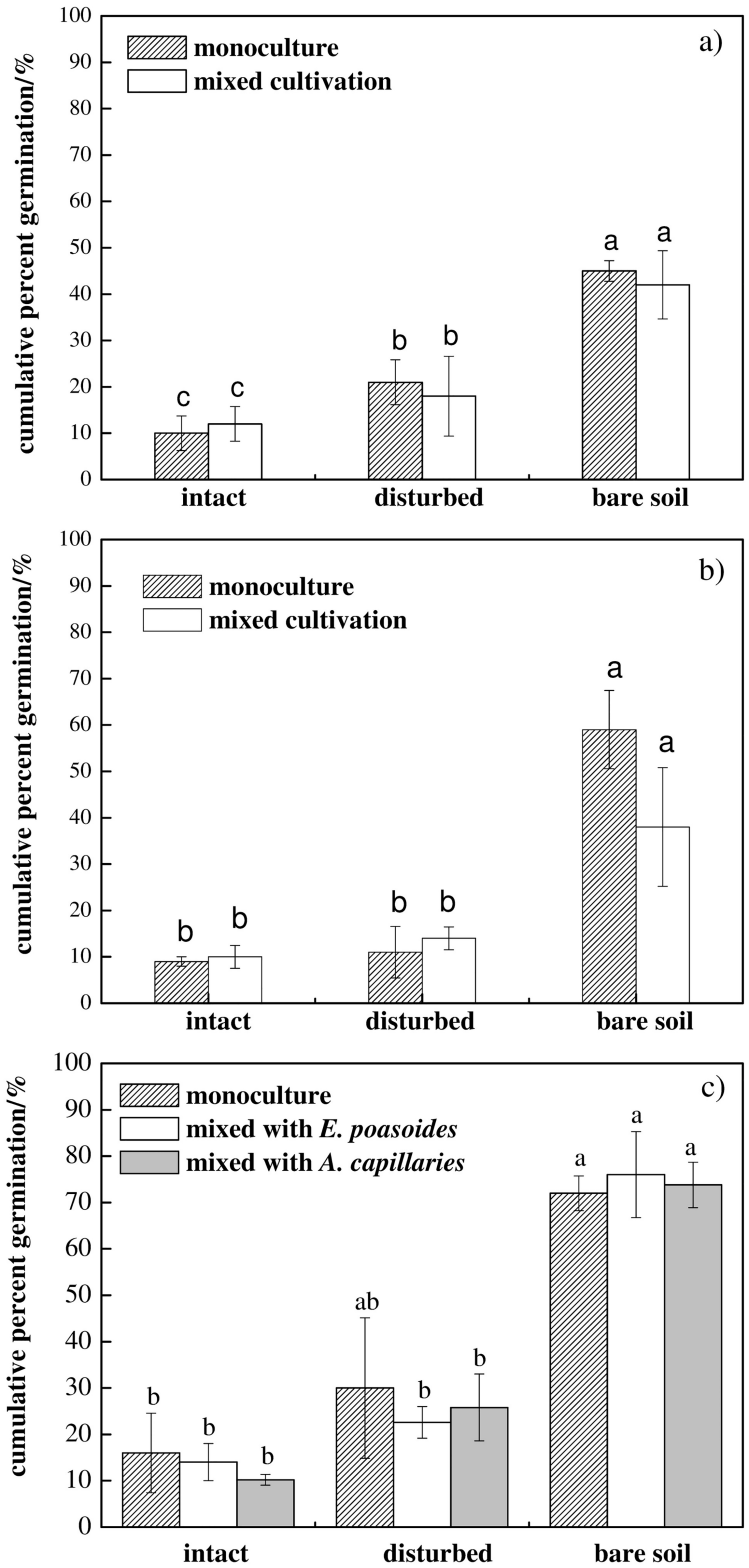
Seed cumulative percent germination *E*. *poaeoides* (a), *A*. *capillaris* (b) and *S*. *glareosa* (c) in different cyanobacterial crust treatments. Values with different lower case letters show significant differences of cumulative percent germination among different experimental treatments at 0.05 levels.

**Fig 2:**
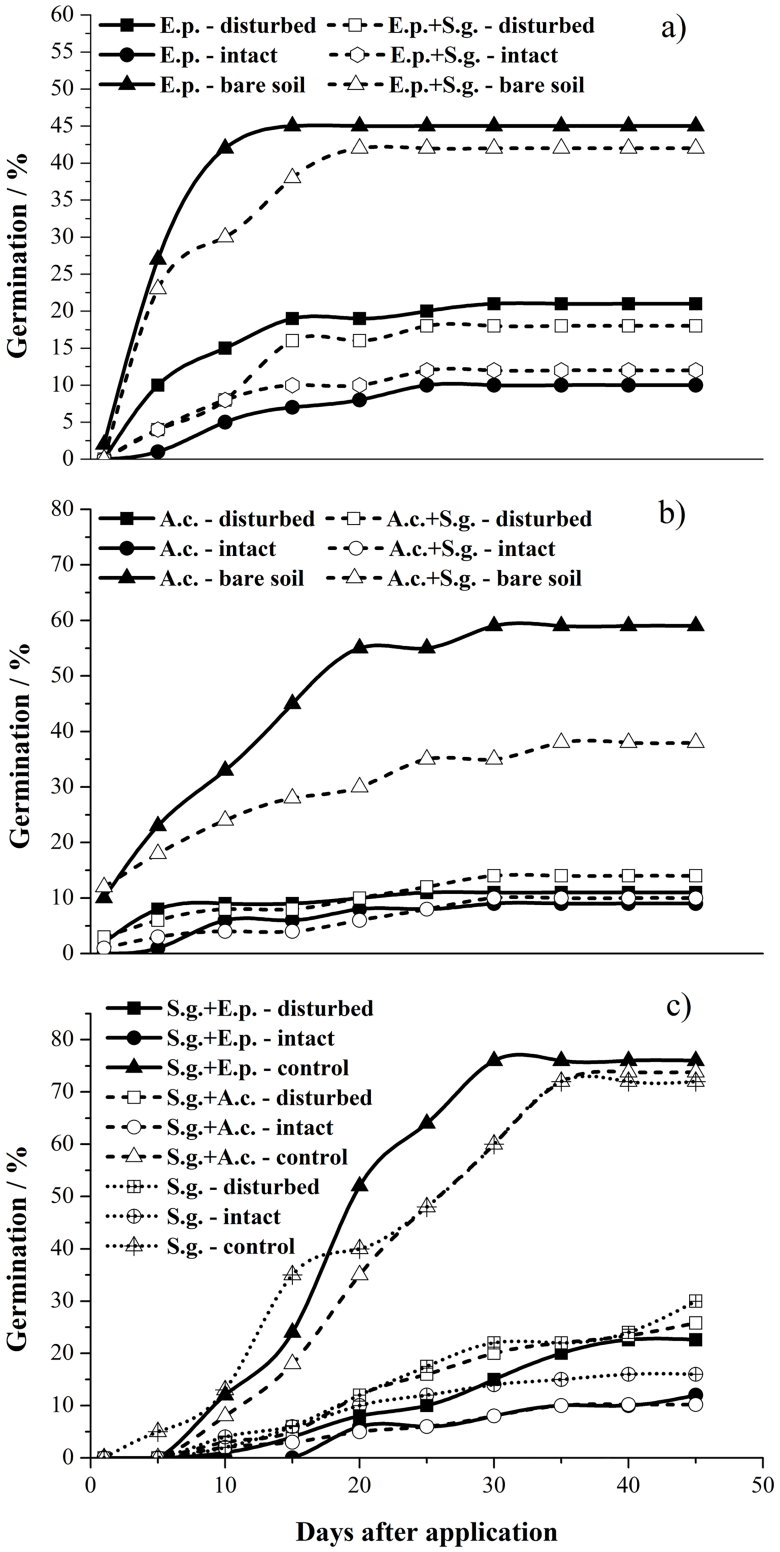
Seeds germination time courses of *E*. *poaeoides* (a), *A*. *capillaris* (b) and *S*. *glareosa* (c) in different cyanobacterial crust treatments. E.p. indicate *E*. *poaeoides* monoculture, E.P. + S.g. indicate *E*. *poaeoides* mixed cultivation with *S*. *glareosa*; A.c. indicate *A*. *capillaris* monoculture, A.c. + S.g.—indicate *A*. *capillaris* mixed cultivation with *S*. *glareosa*; S.g. + E.P. indicate *S*. *glareosa* mixed cultivation with *E*. *poaeoides*, S.g. + A.c. indicate *S*. *glareosa* mixed cultivation with *capillaries* and S.g. indicate *S*. *glareosa* monoculture.

The cumulative percent germination for both native plants, *E*. *poaeoides A*. *capillaris* and exotic grass *S*. *glareosa*, followed the pattern: bare soil > disturbed crust > intact crust (Figs 1 and 2). Differences in cumulative percent germination among the three crust treatments were significant for *E*. *poaeoides* (*P*<0.001). The lowest seed cumulative percent germination (10%) occurred in intact cyanobacterial crusts and the highest (45%) in bare soil (sandy soil) (Figs 1 and 2). For *E*. *poaeoides*, cumulative percent germination was also higher in the disturbed (21%) than in intact cyanobacterial crusts. Results of the multiple comparison suggested that seed cumulative percent germination of *A*. *capillaris* was significantly different in bare soil than in the two crust treatments (bare soil versus intact crust at *P*<0.01, and bare soil versus disturbed crust at *P*<0.001, respectively), but there were no significant differences between disturbed and intact crust treatments (*P* = 0.152).

Cyanobacterial crusts treatments had a significant main effect on the cumulative percent germination of the exotic grass *S*. *glareosa* (*P*<0.001), but the species combination (*P* = 0.528) or the interaction of crust treatments × species did not affect the cumulative percent germination (*P* = 0.190; Table 1). The highest cumulative percent germination occurred in bare soil, followed by disturbed crust, and intact crust for *S*. *glareosa* in monoculture and in mixed-cultivation combinations (Fig 1, S1 Table).

**Table 1:** Results of a two-way ANOVA of cumulative percent germination and shoot biomass of *S*. *glareosa* in different experiment treatments.

	Source of Variance	Type Ⅲ SS	Df	Mean Squares	F	Sig.
**Cumulative percent germination / %**	Corrected model	10378.051^a^	8	1297.256	9.125	.000
	Intercept	64730.918	1	64730.918	455.311	.000
	Crust Treatments	9270.826	2	4635.413	32.605	.000
	Species Combinations	184.889	2	92.445	.650	.528
	Treatments × Species	922.336	4	230.584	1.622	.190
	Error	5118.070	36	142.169		
	Total	80227.038	45			
	Corrected Total	15496.121	44			
**Shoot biomass /mg**	Corrected Model	43.533^b^	8	5.442	1.891	.092
	Intercept	389.243	1	389.243	135.244	.000
	Crust Treatments	29.568	2	14.784	5.137	.011
	Species Combinations	1.522	2	.761	.264	.769
	Treatments × Species	12.443	4	3.111	1.081	.380
	Error	103.611	36	2.878		
	Total	536.387	45			
	Corrected Total	147.144	44			

^a:^ *R*^2^ = 0.541,

^b:^ *R*^2^ = 0.138

In combination with seed germination time courses (Fig 2) and the time to 50% cumulative germination (T50) (Table 2), we analyzed the effects of cyanobacterial crust on the speed of seed germination of exotic plants and native plants. For *E*. *poaeoides*, seeds on bare soil tended to germinate faster (monoculture 4.11±0.51 and mixed 4.55±2.24 days) than on intact (monoculture 10.33±1.94 and mixed 10.77±2.55 days) and disturbed cyanobacterial crust (monoculture 6.84±0.97 and mixed 7.66±2.3 days), no matter whether it growth with or without exotic plant. The time to 50% cumulative germination (T50) of *A*. *capillaris* have no significant difference between disturbed crust and bare soil (*P* > 0.05). In contrast, intact cyanobacterial crust significantly delayed *A*. *capillaris* seeds germinated, which took 13±3.21 and 17.88±2.16 days for monoculture and mixed treatments, respectively. For the exotic species, T50 values of intact and disturbed crusts were no statistically significant (P > 0.05), but seeds on bare soil germinated faster than that on intact and disturbed crust, which took 16.01±1.02, 15.44±2.03 and 15.44±1.69 days for monoculture, mixed with *E*. *poaeoides* and mixed with *A*. *capillaris* treatments, respectively (Table 2).

**Table 2:** The time to 50% cumulative germination (T50) of exotic and native plants in different treatments.

species	treatments	intact	disturbed	sand
***E*. *poaeoides***	monoculture	10.33±1.94a	6.84±0.97b	4.11±0.51c
	mixed with *S*. *glareosa*	10.77±2.55a	7.66±2.3b	4.55±2.24c
***A*. *capillaris***	monoculture	13±3.21a	5.22±2.13b	6.11±1.75b
	mixed with *S*. *glareosa*	17.88±2.16a	7.66±1.6b	5.88±2.12b
***S*. *glareosa***	monoculture	21.44±2.31a	22.27±1.65a	16.01±1.02b
	mixed with *E*. *poaeoides*	20.11±1.74a	20.63±2.31a	15.44±2.03b
	mixed with *A*. *capillaris*	23.22±2.18a	20.24±2.07a	15.44±1.69b

Values with different lower case letters show significant differences of T50 among different experimental treatments at 0.05 levels.

### Influence of cyanobacterial crusts on the biomass

**Fig 3:**
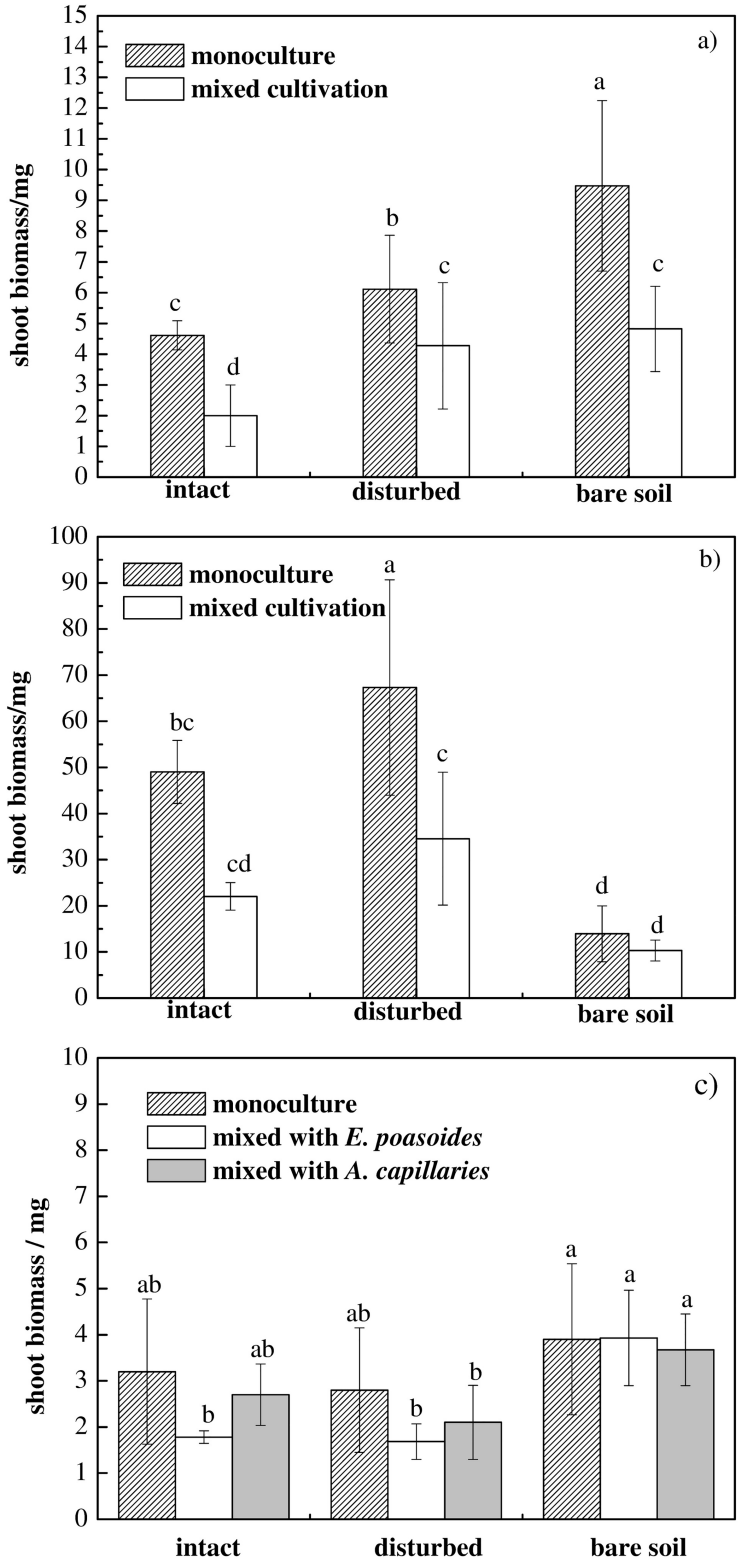
Individual biomass of *E*. *poaeoides* (a), *A*. *capillaris* (b) and *S*. *glareosa* (c) comparison between monoculture and mixed cultivation. Values with different lower letters show significant differences of individual biomass among different experimental treatments at 0.05 levels. Individual biomass denoted average value of all plant biomass in a pot.

Shoot biomass of *E*. *poaeoides* tended to increase with a decrease in the cover of cyanobacterial crusts (Fig 3). Compared to the monoculture of *E*. *poaeoides*, shoot biomass was significantly lower in *E*. *poaeoides* mixed with the exotic grass *S*. *glareosa*. For *A*. *capillaris*, the highest shoot biomass occurred in the disturbed crust treatment when they were monoculture. There are also significant differences between intact crust and bare soil in both the monoculture and the mixed trials (P<0.001). However, results of the two-way ANOVA showed no significant main effect of crust treatments on shoot biomass of *A*. *capillaris* (*P* = 0.111, S2 Table). For the two native plants, there was a significant two-way interaction of crust × species on the shoot biomass (*E*. *poaeoides*: *P* = 0.016; *A*. *capillaris*: *P* = 0.001).

For *E*. *poaeoides*, the main effect of cyanobacterial crusts on shoot biomass was significant (*P*<0.05), but the main effect of exotic grass on its growth was not significant (*P* = 0.548, S2 Table). However, the two-dimensional interaction of cyanobacterial crusts treatments × species combination was significant for shoot biomass (*P*<0.05). For *A*. *capillaris*, the main effect of the crust treatment or the species combination was not significant for shoot biomass of *A*. *capillaris* (*P* = 0.111, *P* = 0.242, S2 Table). Similar to *E*. *poaeoides*, the two-way interaction of crust treatment × species combination was significant (*P*<0.001). In view of the significant two-way interaction, a simple-effect analysis was used to evaluate shoot biomass differences between monoculture and mixed cultivation. The results showed that the shoot biomass of the two native plants markedly decreased when they were mixed with the exotic grass compared to their growth monoculture, except for *A*. *capillaris* in bare soil (Fig 3).

For *S*. *glareosa*, results of the two-way ANOVA indicated that the main effect of the crust treatment on individual biomass was significant, but the species combination was not (Table 1). Shoot biomass of *S*. *glareosa* was higher in bare soil than in intact and disturbed crusts in all *S*. *glareosa* species combinations (monoculture, mixed cultivation with *E*. *poaeoides* and *A*. *capillaris*) (Fig 3, S1 Table).

## Discussion and conclusions

Our study suggested that the cyanobacterial crust treatments had significant effects on the germination of two plants species native to the re-vegetated sand dunes of the Tengger Desert. As shown in other studies, germination rate was related to the available contact area between seed and soil [26,72]. Cyanobacterial crusts decreased the roughness of the soil surface, and inhibited seed germination of plants by reducing the contact area between seed and soil. Differences in cumulative percent germination between native and exotic plants may be related to differences in seed morphology. When the trypanophorous diaspores of *S*. *glareosa* seeds become buried by hygroscopic movement of awns [73,74], the awns can absorb and transport moisture to the embryo at the seed base [75].

The physical effects of cyanobacteria crusts make it harder for *S*. *glareosa* seeds to reach moist soil than in bare soil. Seeds of the two native plants in this study cannot enter into a soil pore without the help of an external influence (e.g. rain, wind) even if large contact area with the soil is available. Therefore, native species did not have a competitive advantage when growing together with exotic species due to the difference in seed morphology. Because *S*. *glareosa* exhibits high ecological adaptability and dispersion rates, this exotic grass can maintain high population numbers in new habitats [76]. In fact, population expansion of invasive species in new habitats is the fundamental reason for significant damage to the invaded ecosystems [77,78]. Cumulative percent germinations of the exotic grass and two native plants were significantly lower in intact and in disturbed cyanobacterial crusts than in bare soil. The results showed higher germination on bare soil than disturbed crust. This may be partly attributing to that all treatments could gain enough distilled water every day. As Li et al. [32] concluded that an increase in precipitation may override any positive influences of crusts on the germination of vascular plants in temperate desert regions. Another research also suggested that the effects of BSCs on seed germination of plant species were variable under both moist and dry conditions [34,35]. Moreover, the other reason may be that seeds on bare soil have bigger contact area with soil than that on disturbed crusts. Although germination of two native and one exotic grass were inhibited by cyanobacterial crust (intact and disturbed crust), the inhibition of cyanobacterial crust was stronger on germination of *S*. *glareosa* than of *E*. *poaeoides* and *A*. *capillaris* (Fig 3). Similar results were observed in other studies which showed that the germination and establishment of an annual exotic grass, *Bromus tectorum*, was inhibited by BSCs [26,79,80]. Crisp [81] also found that BSCs had an inhibiting effect on seed germination and establishment of the annual invasive grass *Schismus*. This response may be associated with chemical substances secreted by cyanobacteria under conditions of sufficient soil water, which can inhibit seed germination of invasive plants [82].

In our study, the lowest shoot biomass of *E*. *poaeoides* and *S*. *glareosa* was found in soil covered by intact cyanobacterial crusts (Fig 3). This seems inconsistent with previous studies that positive effects of BSCs on vascular plants [30,83]. For instance, Zhang and Nie [84] found the presence of BSCs can increase biomass accumulation of herbaceous species in the desert of northwestern China. Higher herbaceous and woody plant cover and biomass were observed on the biocrust-stabilized dunes in the Negev desert [85]. We attribute the reason to that the time between germination and harvest is shorter for the seeds that germinated on the crust (Fig 2, Table 2). Seeds on the bare soil germinate earlier and have longer growth time, so they accumulate more substance and have higher biomass. However, our results also showed positive (*A*. *capillaris*) and negative (*E*. *poasoides*) effects on individual biomass of the two native plants. Because T50 values of *E*. *poasoides* seeds have significant different among intact, disturbed crust and bare soil treatments. While T50 values of *A*. *capillaris* were no statistically significant between disturbed crusts and bare soil. Additionally, other studies have shown that disturbance may temporarily increase available nutrients [5,27]; this may explain the higher biomass of *A*. *capillaris* in disturbed than in intact crusts and in bare soil. But seeds of *A*. *capillaris* on intact crusts germinated too late to gain enough growth time, so the lowest biomass were found on the intact cyanobacterial crusts (Fig 3). These results showed that cyanobacteria have species-specific effects on the growth of native and exotic plants [37,86,36].

Characteristics of exotic species, possessed extreme competitiveness for resources, high adaptability to the environment, and high resistance to disturbance, play an important role in their success as invasive plants [87,88]. The exotic annual grass, *S*. *glareosa*, acquired these features in the process of long-term adaptation to arid environments which facilitates the plant’s expansion to new habitats including sandy soils [69,89]. *S*. *glareosa* can acquire more water than the two native plants through the allocation of biomass to its intricate root system [89]. Therefore, individual biomass of both *E*. *poaeoides* and *A*. *capillares* decreased in mixed cultivation treatment compared with monoculture (Fig 3). Nevertheless, the exotic plant species can affect native plants growth through combined different cyanobacterial crust (intact, disturbed crust and bare soil). This indicated that the effect of *S*. *glareosa* on native plants depended on whether the crust was intact or disturbed. Our results suggested that intact cyanobacterial crusts, an important biotic component of dryland ecosystems, can restrict germination vascular plant species. The inhibitory effect of cyanobacterial crusts on germination also decreased the germination of the exotic grass, annual *S*. *glareosa*. Therefore, the greenhouse tests may suggest that BSCs (cyanobacteria-type) would constitute the biotic resistance of arid ecosystems, and play a positive role in the ability of this ecosystem to withstand invasions of exotic plants.

In conclusion, an increasing rate and intensity of anthropogenic disturbances result in an increased rate of exotic species invasions in arid regions. BSCs are the most important biotic factors in arid ecosystems and show promise as potential inhibitors of exotic plant invasions. Under greenhouse conditions, cyanobacterial crusts can reduce germination of the exotic grass and two native plants, which could be partly attributed to the formation of a physical barrier. Namely, this may indicated that BSCs (cyanobacteria-type) under filed conditions form a natural biological barrier which could prevent plant invasions in re-vegetation areas. Cyanobacterial crusts may slow down colonization of an area by particular exotic plant species by reducing seed germination. This would tend to maintain more diverse plant communities and contribute to the formation of clumped vegetation patterns [26,90]. Further work is needed to investigate the ecological significance of the results presented. However, various disturbances to BSCs may result in increased rates of exotic species invasions in arid regions. Therefore, protection of BSCs can enhance sustainability and stability of this fragile ecosystem.

## Supporting information

S1 Table Seed germination rate and biomass of S. glareosa in different conditions. (DOCX)

S2 Table Two-way ANOVA of individual biomass of E. poaeoides and A. capillaries in different cyanobacteria crusts treatments and species combinations. (DOCX)
