# Supplementary material for: Effect of biological soil crusts on seed germination and growth of an exotic and two native plant species in an arid ecosystem
Source: PLoS One. 2017 Oct 4;12(10):e0185839. doi: 10.1371/journal.pone.0185839 (PMC5627943; doi:10.1371/journal.pone.0185839)
Supplement: S1 Table — (DOCX) [file pone.0185839.s001.docx]

**S1 Table** Seed germination rate and biomass of *S. glareosa* in different conditions

| Species | Crust treatments | Germination rate % | Individual aboveground biomass /mg |
| --- | --- | --- | --- |
| *S.* *glareosa* (S+E) | Intact | 14±3.99 | 1.78±0.14 |
|  | disturbed | 23±3.41 | 1.68±0.39 |
|  | bare soil | 76±9.26 | 4.93±1.03 |
| *S. glareosa* (S+A) | Intact | 32±8.59 | 2.70±0.66 |
|  | disturbed | 40±15.14 | 2.10±0.80 |
|  | bare soil | 62±3.74 | 3.67±0.77 |

Note: *S. glareosa* (S+E) and *S. glareosa* (S +A) denote *S. glareosa* mixed with *E. poaeoides* or *A. capillaries*, [respectively](javascript:void(0);)**.**
