# Supplementary material for: Effect of biological soil crusts on seed germination and growth of an exotic and two native plant species in an arid ecosystem
Source: PLoS One. 2017 Oct 4;12(10):e0185839. doi: 10.1371/journal.pone.0185839 (PMC5627943; doi:10.1371/journal.pone.0185839)
Supplement: S2 Table — (DOCX) [file pone.0185839.s002.docx]

**S2 Table** Two-way ANOVA of shoot biomass of *E. poaeoides* and *A. capillaries* in different cyanobacteria crusts treatments and species combinations

|  | **Source of Variance** | **Type Ⅲ SS** | **Df** | **Mean Squares** | **F** | **Sig.** |
| --- | --- | --- | --- | --- | --- | --- |
| **E. poaeoides** | Corrected model | 83.369a | 5 | 16.674 | 4.156 | .007 |
|  | Intercept | 385.776 | 1 | 385.776 | 96.157 | .000 |
|  | Crust Treatments | 42.262 | 2 | 21.131 | 5.267 | .013 |
|  | Species Combinations | 1.493 | 1 | 1.493 | .372 | .548 |
|  | Treatments × Species | 39.615 | 2 | 19.807 | 4.937 | .016 |
|  | Error | 96.287 | 24 | 4.012 |  |  |
|  | Total | 565.433 | 30 |  |  |  |
|  | Corrected Total | 179.656 | 29 |  |  |  |
| **A. capillaries** | Corrected Model | 106.000b | 5 | 21.200 | 5.278 | .002 |
|  | Intercept | 248.131 | 1 | 248.131 | 61.779 | .000 |
|  | Crust Treatments | 19.419 | 2 | 9.710 | 2.417 | .111 |
|  | Species Combinations | 5.772 | 1 | 5.772 | 1.437 | .242 |
|  | Treatments × Species | 80.809 | 2 | 40.405 | 10.060 | .001 |
|  | Error | 96.395 | 24 | 4.016 |  |  |
|  | Total | 450.526 | 30 |  |  |  |
|  | Corrected Total | 202.395 | 29 |  |  |  |
| a: R^2^=0.352, b:R^2^=0.425 | | | | | | |
